# JAMI: a Java library for molecular interactions and data interoperability

**DOI:** 10.1186/s12859-018-2119-0

**Published:** 2018-04-11

**Authors:** M. Sivade (Dumousseau), M. Koch, A. Shrivastava, D. Alonso-López, J. De Las Rivas, N. del-Toro, C. W. Combe, B. H. M. Meldal, J. Heimbach, J. Rappsilber, J. Sullivan, Y. Yehudi, S. Orchard

**Affiliations:** 1 European Bioinformatics Institute (EMBL-EBI), European Molecular Biology Laboratory, Wellcome Genome Campus, Hinxton, CB10 1SD UK; 2 Cancer Research Center (CiC-IBMCC, CSIC/USAL/IBSAL), Consejo Superior de Investigaciones Científicas (CSIC) and Universidad de Salamanca (USAL), 37007 Salamanca, Spain; 3 Wellcome Trust Centre for Cell Biology, School of Biological Sciences, University of Edinburgh, Edinburgh, EH9 3BF UK; 4 Bioanalytics, Institute for Biotechnology, Technische Universität Berlin, 13355 Berlin, Germany; 5 Cambridge Systems Biology Centre, University of Cambridge, Cambridge, UK; 6 Department of Genetics, University of Cambridge, Cambridge, UK

**Keywords:** Molecular interactions, Protein-protein interaction, Protein complexes, Data standards, HUPO-PSI, PSI-MI

## Abstract

**Background:**

A number of different molecular interactions data download formats now exist, designed to allow access to these valuable data by diverse user groups. These formats include the PSI-XML and MITAB standard interchange formats developed by Molecular Interaction workgroup of the HUPO-PSI in addition to other, use-specific downloads produced by other resources. The onus is currently on the user to ensure that a piece of software is capable of read/writing all necessary versions of each format. This problem may increase, as data providers strive to meet ever more sophisticated user demands and data types.

**Results:**

A collaboration between EMBL-EBI and the University of Cambridge has produced JAMI, a single library to unify standard molecular interaction data formats such as PSI-MI XML and PSI-MITAB. The JAMI free, open-source library enables the development of molecular interaction computational tools and pipelines without the need to produce different versions of software to read different versions of the data formats.

**Conclusion:**

Software and tools developed on top of the JAMI framework are able to integrate and support both PSI-MI XML and PSI-MITAB. The use of JAMI avoids the requirement to chain conversions between formats in order to reach a desired output format and prevents code and unit test duplication as the code becomes more modular. JAMI’s model interfaces are abstracted from the underlying format, hiding the complexity and requirements of each data format from developers using JAMI as a library.

## Background

Molecular interaction data is crucial to the study and understanding of the molecular biology of a cell. These data are large and complex, but the creation of a standardised data interchange format (PSI-MI XML) allowed easier access, enabling users to merge data from disparate resources and encouraging the development of tools and software that facilitated network visualisation and analysis. Version 1.0 [1] of the format only allowed a relatively simple description of protein interactions but as the data grew, limitations of the original format were identified, and an updated version, PSI-MI XML2.5 [2], was released in 2007. It allows the description of interactions between molecules other than proteins, and enables the detailed capture of both experimental context and the constructs used in each assay. This version of the interchange format is still widely used to capture experimental data, but the need to describe more abstract concepts has recently resulted in the release of PSI-MI XML 3.0 [3]. PSI-MI XML3.0 allows the capture of details of cooperative or allosteric binding sites, the composition of protein complexes taken from multiple publications, and more complex data types such as dynamic interaction networks that change with time or with concentration of agonist. A simpler tab-delimited representation of molecular interaction data has also been available since 2007 but this has also grown in complexity in response to user requests, and MITAB2.5, 2.6 and 2.7 are now all available [2]. Additionally, at the 2017 HUPO-PSI workshop, the Molecular Interaction workgroup decided the newly developed MI-JSON will be its recommended protocol for serving interaction data to web pages and visualisation tools.

PSI-MI XML, MITAB and MI-JSON are all capable of holding the same data, in differing degrees of detail, and are all annotated using a single shared controlled vocabulary but exist to serve different user groups. The XML format is largely used by software developers and database managers, the MITAB by biologists interested in simple binary representation and the MI-JSON for visual representation. Updating any data format necessitates changes to many dependent systems. A broad range of software, including curation, editing, export, visualisation, validation and analysis packages use the PSI-MI formats to access and manipulate the data and consequently need to be updated with every format update. Format updates add complexity to existing software packages, as the programs need to be extended to utilise the new version whilst still continuing to support those already existing and widely-used. These software and standards are consumed by a diverse group of organisations with different levels of resources, ranging from PhD students in small research groups to data pipeline specialists in pharmaceutical or bioinformatics companies. Potentially some groups may end up using legacy standards and software for many years simply because they do not possess the skills, time, or budget to update their software.

Supporting such diverse needs is time and resource intensive, yet securing funding for software maintenance is challenging [4]. Each new data format is useful and must be maintained, but each update generates a new library, with duplicated code, requiring parallel testing and generating its own bugs. In summary, while new formats meet genuine need, they also result in an expensive cascade of changes to software and tools.

The JAMI (Java Molecular Interaction framework) library was developed, using an object-orientated approach, to address these concerns. JAMI can import, inter-convert and re-export molecular interaction data in a variety of formats and versions. The software has been designed to ensure that modules to read/write new format types can easily be written and added to the library, thus providing a single change-resilient software component to handle all molecular interaction data. It is generally intended that the JAMI library will be used within a Java application, rather than being made available as an API, but users could look to develop a programmatic interface using the JAMI framework, if required. Given the change-resilient remit of the JAMI framework, it was necessary to ensure that JAMI can handle multiple use cases. It needs to concurrently support legacy data models, contemporary data models, and any new changes required in the future, as interaction data becomes ever more sophisticated in its nature. For this reason, the data model was deliberately kept flexible enough to expand, with all classes being interfaces with a default implementation. Implementations may be added, edited or removed if necessary over time. Main entities in the data model include Complex, Interaction, Entity, Participant, and Publication - interfaces with a default implementation and format-specific overloaded behaviours. For example, PSI-XML 2.5 [2] allowed experiment descriptions to contain either a cross-reference to a Publication object, or directly contain a list of attributes such as author and journal, whereas in XML 3.0, it is possible to associate both of these data members with an experiment [3]. Since the Publication and XML export classes are only interfaces, exporting the two different types of Publication can be handled by the same software, with implementation classes reconciling the two XML versions.

When included as a library in bioinformatics software, JAMI hides the complexity of supporting multiple data formats. It facilitates data import, integration and analysis, simplifying software development by offering a single API. JAMI also eases the creation of new interchange formats, like JSON-LD or RDF. Additional formats can be added once to JAMI and are then supported in multiple software packages with little effort. Similarly, JAMI prevents code duplication - each of these software sources drawing from JAMI now share code, ensuring less effort is put into the development of multiple XML/MITAB parsing modules.

## Implementation

**Fig. 1:**
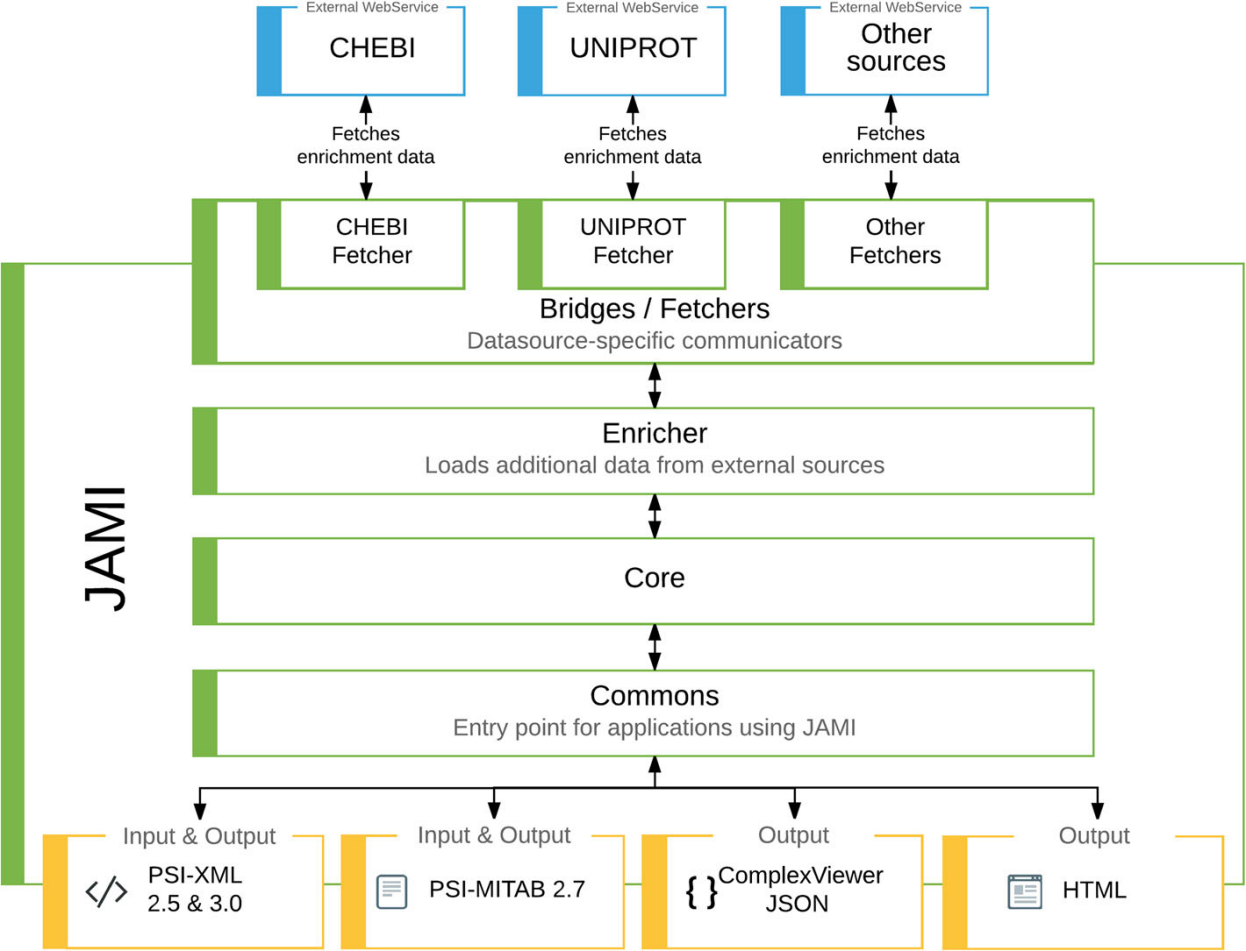
JAMI internal architecture and input-output formats, including web service data

Figure 1 shows the overall architecture of the JAMI library. JAMI is implemented in Java, using Maven for distribution and dependencies (https://www.ebi.ac.uk/intact/maven/nexus/content/repositories/ebi-repo/psidev/psi/mi/jami/)..The code is available under the Apache 2.0 licence, and is available on GitHub [5]. The architecture is highly modular, driven by the anticipated need to modify or add input and output types in the future, without affecting the overall function or capabilities of the framework. Unit tests cover core functionality, ensuring that code changes do not result in regression (returning bugs) and that input/output formats and behaviours remain consistent even when code is changed. Travis CI (https://travis-ci.org/), performs continuous integration, automatically running the test suite whenever the codebase is changed.

### JAMI-Core and JAMI-commons

JAMI-Core forms the foundation of JAMI, comprising of standalone modules for each data input/output type that JAMI is capable of handling, alongside appropriate listeners and factories to instantiate JAMI’s classes. For software using JAMI internally, JAMI-Commons serves as the default entry point, functioning as a thin code-loading helper to import the XML and MITAB input/output modules, along with all relevant code dependencies. Dependencies are limited where possible to prevent potentially disruptive updates from third parties.

JAMI-Core is also the home of code that handles spoke and matrix expansion. Spoke or matrix expansion is used to convert interactions with more than two participants into multiple binary interactions - this is frequently needed as many tools only operate on binary interactions.

### Data input/output types

In addition to both reading and writing MITAB2.5, 2.6 and 2.7 and PSI-XML (2.5 and 3.0), JAMI is also able to output HTML and MI-JSON (Table 1). The HTML format is intended for quick human consumption via a web browser, and is currently hardwired in JAMI to produce a consistent layout and appearance although further CSS extensibility is planned in the future, if there is user demand. The MI-JSON output is designed for ease of use with JavaScript-based client applications, such as the ComplexViewer [6] described later in the results section. As described above, JAMI is designed to be readily extensible. For example, components are currently being created to support semantic web formats (i.e. RDF), which are a base to realise interoperable linked data and an extension to enable protein complexes described in PSI-MI XML3.0 to be converted into an SBGN representation is also being developed (https://github.com/MICommunity/mi-sbgn). A JAMI-JSON-LD module is also in preparation to enable export to Wikidata.

**Table 1:** Input and output formats currently provided by the JAMI library

Format	Input?	Output?
PSI-XML (2.5 and 3.0)	Yes	Yes
MITAB 2.7	Yes	Yes
MI-JSON	No	Yes
HTML	No	Yes

### Data enrichment

JAMI’s enricher package allows known interactors sparsely annotated with little descriptive information to be enriched with additional data accessed from external sources. An example of this, as shown in blue at the top of Fig. 1, demonstrates fetching additional data from UniProtKB [7] to enrich protein interactors and ChEBI [8] for small molecules.

The enrichment package communicates with external web services via a suite of modular web service-specific fetcher packages within the JAMI-Bridges package. Many of these sources are biomedical ontologies and a module (JAMI-OLS) has been developed to import data via the Ontology Look-up Service (OLS), thus giving the user access to the 204 ontologies (December 2017) available through the OLS API. Separating the fetcher/bridges packages from the enricher provides an abstraction layer that ensures external changes, such as adding or removing enrichment sources, cannot unintentionally affect the entire software architecture.

### Data model

Given the change-resilient remit of the JAMI framework, it is necessary to ensure that JAMI can handle multiple current use cases. It needs to concurrently support legacy data models, contemporary data models, and any new changes enacted. For this reason, the data model was deliberately kept flexible enough to expand, with all classes being interfaces with a default implementation. Implementations may be added, edited or removed if necessary over time.

## Results/discussion

The Proteomics Standards Initiative (PSI) has developed and actively promotes the use of open standard data formats and has a proven track record in developing robust, pluggable programming interfaces to address the issue of data being made available in a range of formats [9].

The JAMI library has been created to address this problem in the field of molecular interactions and has already been adopted by a number of resources which process, import, export and/or visualise interaction data. We describe below a number of implementations and use these to exemplify the utility and flexibility of the JAMI framework.

## ComplexViewer

**Fig. 2:**
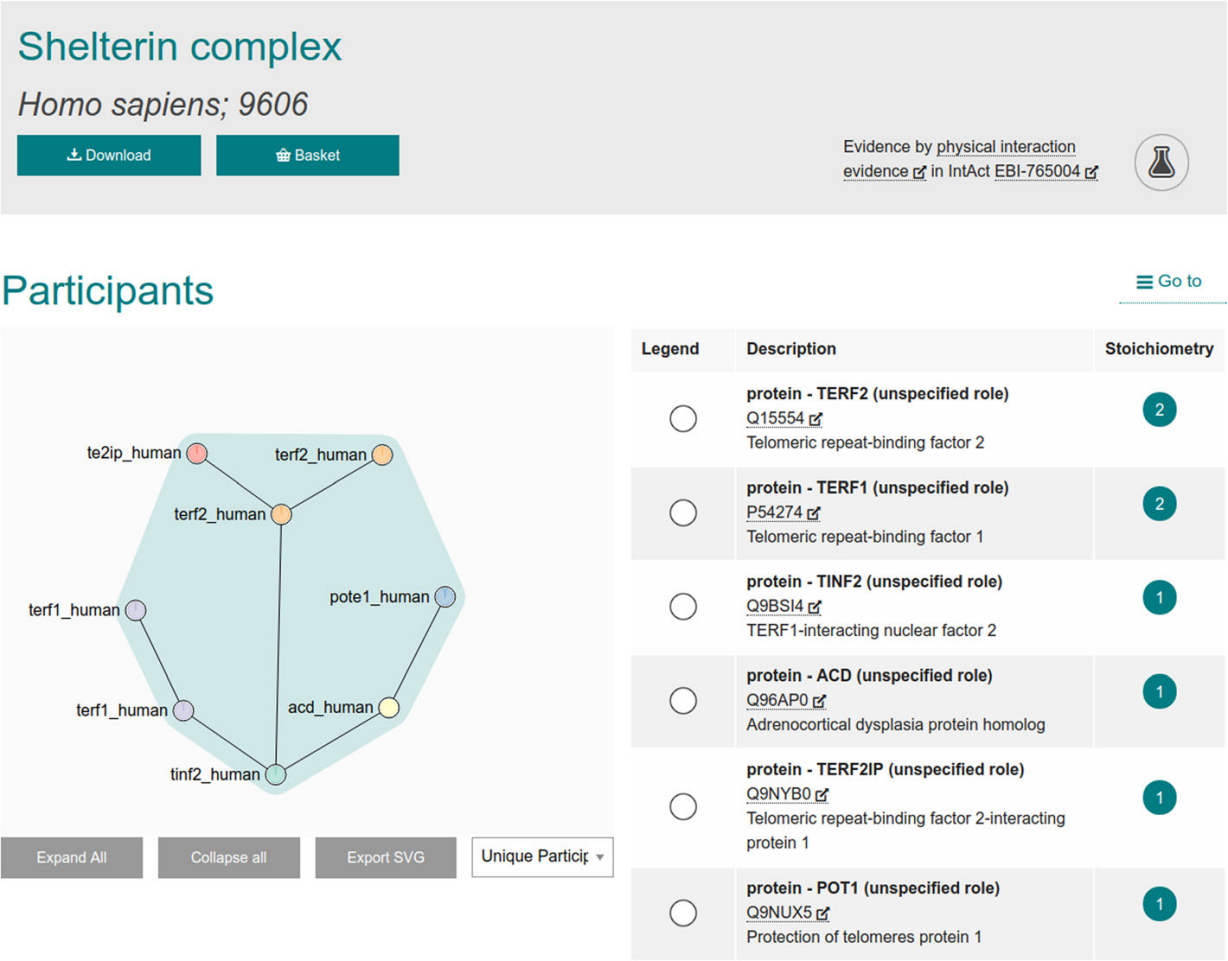
The ComplexViewer designed by the Rappsilber lab showing the collapsed and b. the expanded view of complex participants

**Fig. 3:**
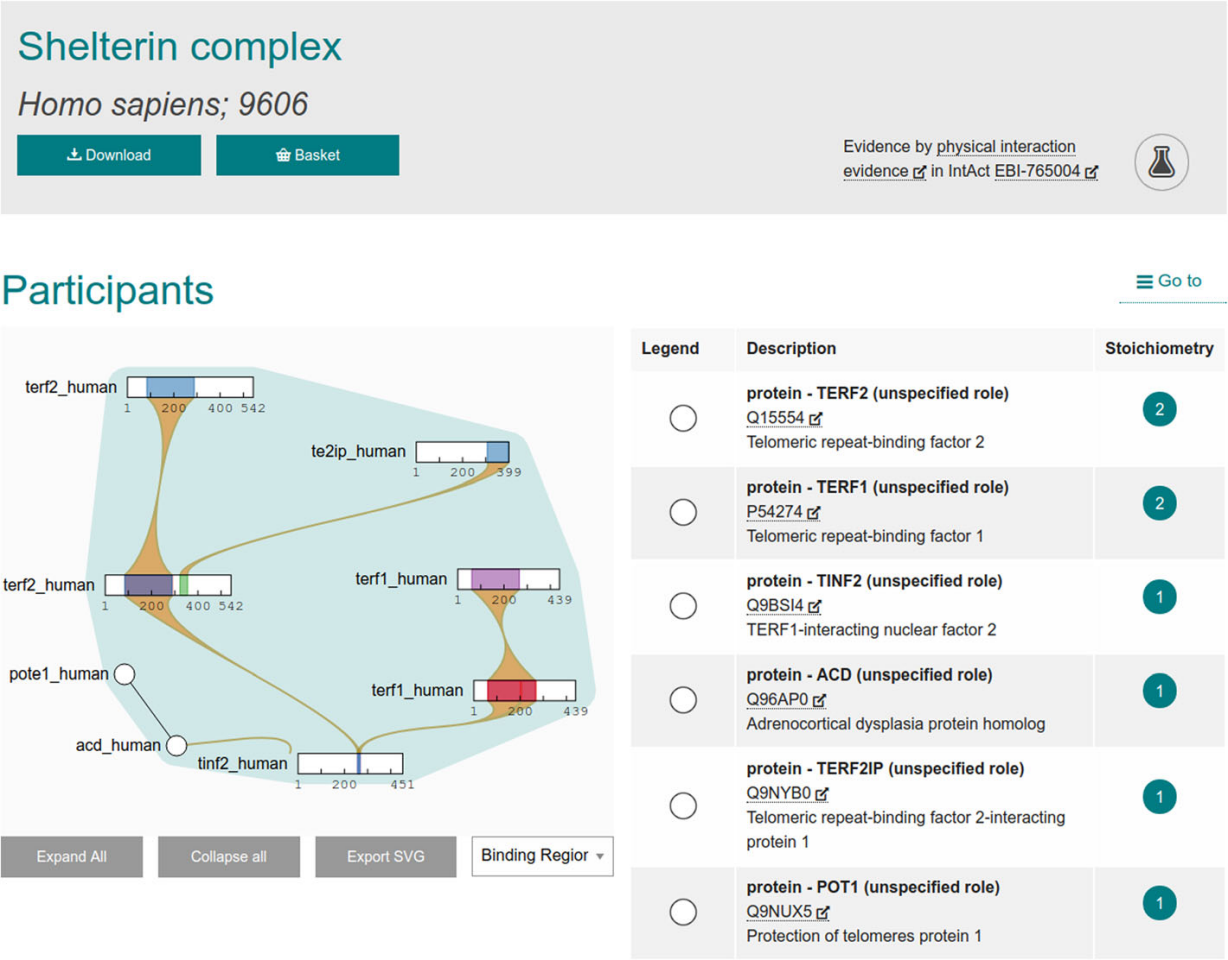
The ComplexViewer designed by the Rappsilber lab showing the expanded view of complex participants

The ComplexViewer pictured in Figs. 2 and 3 is a powerful visualisation tool which can show its users exactly how the molecules in complex interactions interact with each other, with interactive controls to allow data exploration [6]. Unlike other network viewers, this interaction viewer details the binding sites and regions, when known, and enables the topology of a macromolecular complex to be fully represented. Inter-molecular connections can be demonstrated down to the amino acid level, in the case of proteins, or shown as binding domains or interfaces. The JAMI-JSON module in JAMI is used to produce the MI-JSON file required by this specialist visualisation software. ComplexViewer is open source and hosted on GitHub under the Apache 2.0 licence and has been integrated into the Complex Portal [10], HumanMine [11] and YeastMine [12] data resources.

## InterMine

The biological data warehouse InterMine [11] provides another concrete example of how JAMI can be used to input and output molecular interaction data. InterMine is organism-agnostic and open source, designed to consolidate discrete data sources with varied data formats into a single database. Data can be accessed via a web application interface or more directly via APIs, with clients available in multiple languages. Efficient and maintainable data import and export is therefore of particular significance to InterMine.

**Fig. 4:**
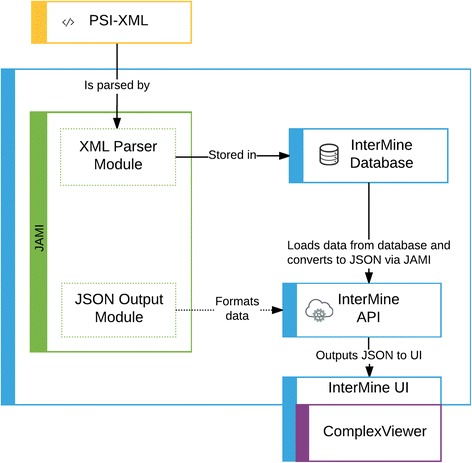
Workflow showing how JAMI library helps InterMine software import and export data

As shown in Fig. 4, InterMine uses the JAMI library to read molecular interaction data in PSI-XML format, retrieved from IntAct [13]. JAMI then translates the interaction data into interaction objects. These interaction objects are parsed by InterMine, changed into InterMine objects and then stored into the database at build time. InterMine’s use of JAMI to import XML removes the need for InterMine to implement multiple PSI-XML version-specific parsers, and future-proofs the InterMine code against any potential new changes in the PSI-XML specifications. InterMine also uses JAMI to export interaction data. In InterMine instances where complex interaction data is loaded, for example HumanMine and YeastMine [11], the ComplexViewer shown in Figs. 2 and 3 is available on the InterMine report page.

As detailed above, the specialist ComplexViewer visualisation software requires a custom MI-JSON data format for input. Once again, InterMine uses the JAMI library to perform this task, by querying the database for data related to the interaction, and transforming it to match the JAMI data model. JAMI then is able to export a MI-JSON file to ComplexViewer.

## IntAct

**Fig. 5:**
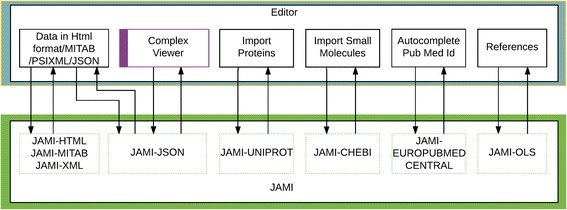
Usage of JAMI in the curation tool (Editor) application of the IntAct molecular interaction database

The IntAct molecular interaction database [13] uses JAMI internally for data format conversions and imports, as well as for writing to its own internal database. The IntAct Editor application is used to curate molecular interactions data, and uses JAMI extensively (Fig. 5), utilising the JAMI-HTML, JAMI-MITAB, JAMI-XML and JAMI-JSON modules for exporting publications, experiments, interactions and complexes in the relevant formats. This application integrates the ComplexViewer described in the previous section, and like the InterMine use case, it is fed by the JAMI-JSON module. Using JAMI-UNIPROT and JAMI-CHEBI, the editor can import proteins from UniProtKB and small molecules from ChEBI. Similarly, the use of a publication’s Pubmed ID allows further details relating to that paper to be imported into the Editor from EuropePubmedCentral via the JAMI- EUROPUBMED CENTRAL module. Ontology terms, e.g. from the Gene Ontology [14] and Molecular Interactions ontologies [15], are fetched from OLS [16] using the JAMI-OLS module. Finally, when IntAct releases curated data into the public database, the JAMI-XML and JAMI-MITAB modules generate PSI-XML and PSI-MITAB files and place them on an FTP server for public access. The IntAct database currently (December 17) contains 800,000 binary interaction evidences from more than 20,000 publications and this data is updated and released on a monthly basis using the JAMI library. The December 2017 release totalled 55GB of data. Over 5000 publication were recently imported into the IntAct database from DIP [17], to centralise the IMEx dataset, and this was performed as a single import operation using a tool reliant on the JAMI modules.

## Complex portal

**Fig. 6:**
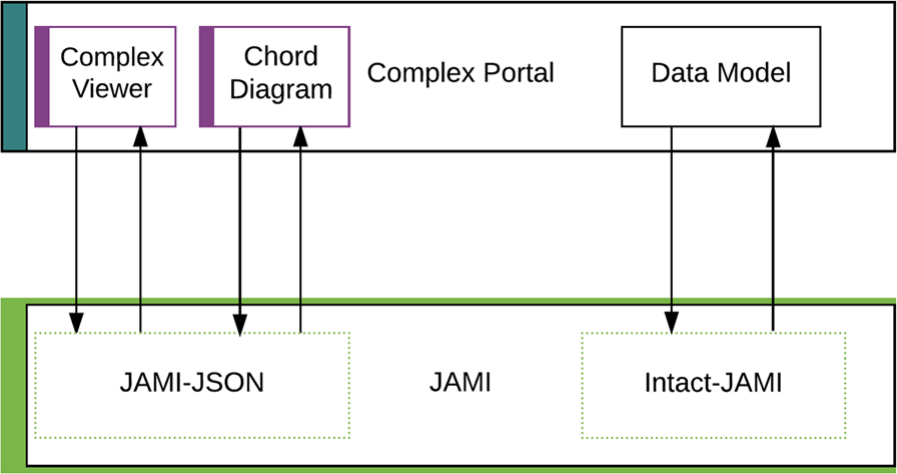
Usage of JAMI in the “Complex Portal” application of the IntAct database

The Complex Portal [9] is a manually curated, encyclopaedic resource of macromolecular complexes from a number of key model organisms, where data is freely available for search and download. Each protein complex entry is stored in the IntAct database but is served to a distinct website (http://www.ebi.ac.uk/complexportal) and the files are written to a separate FTP site, all managed internally by the JAMI library (Fig. 6). The ComplexViewer, described above, is also used in the Complex Portal and utilises JAMI-JSON to provide visualisation of the complex data.

## Chord interaction diagram

**Fig. 7:**
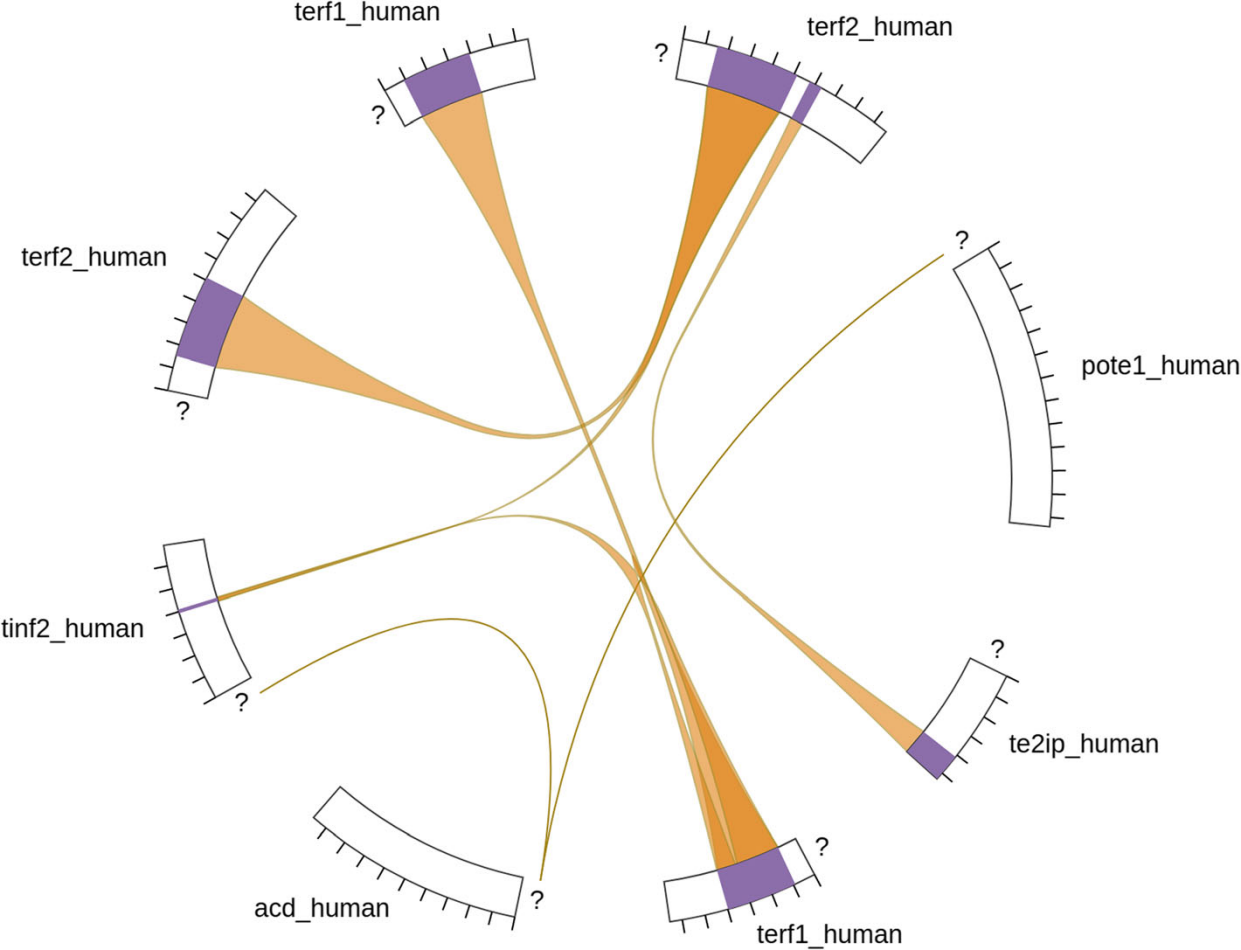
JAMI-JSON driven chord diagram of a protein complex

Figure 7 shows the chord diagram (currently in beta development), which is conceptually similar to the ComplexViewer, drawing upon MI-JSON generated by JAMI to create an alternative visualisation of the interactions in a protein complex. While developed independently of the ComplexViewer, it too uses MI-JSON generated by JAMI as its data model.

## HUPO PSI-MI semantic validator

Molecular interaction files can be either rapidly curated, curated to MIMIx specifications [18] or contain the detailed information captured by members of the IMEx Consortium [19] but, in all cases, need to be semantically valid to enable data exchange and merger. The PSI-MI semantic validator (http://www.ebi.ac.uk/intact/validator) [20] not only checks the XML syntax of a submitted file but also enforces rules regarding the use of an ontology class or CV terms by checking that the terms exist in the resource and that they are used in the correct location of a document. Previously, the validator was only able to validate PSI-PAR and PSI-XML 2.5, but using JAMI it can now also validate MITAB 2.5, 2.6, and 2.7 and PSI-XML 3.0 [3]].

## Agile protein Interactomes DataServer (APID)

APID (Agile Protein Interactomes DataServer) [21] is a database that provides a comprehensive collection of protein interactomes for more than 400 organisms based on the integration of known, experimentally validated protein-protein physical interactions from several primary databases, e.g. BIND [22], BioGRID [23], DIP, HPRD [24], IntAct, and MINT. Construction of the interactomes is done with a methodological approach to report quality levels and coverage over the proteomes for each organism included. The APID algorithm uses a protocol based on JAMI to process all of the PSI-XML formatted data and then uses the JAMI-generated interaction objects in all the workflows which have been subsequently implemented by this resource. It also takes advantage of the ability of JAMI to expand complexes when multiple interactions are detected.

### Future plans

The current implementation of JAMI provides the molecular interactions community with a powerful library to enable the development and long-term maintenance of third-party tools. It enables these formats to be updated and refreshed in response to new data types and resources, and is capable of read/writing all existing version of the PSI-MI XML and MITAB formats and methodologies without obsoleting existing tools and resources. The PSI formats, however, are not the only mechanisms available for exchange of molecular interaction data and we intend to use the robust architecture of JAMI to provide additional read/writers for XGMML, BioPax and RDF. Additionally, we will use the JAMI framework to improve users’ ability to merge data from different resources, improving the existing MImerge software [25], and use the ability to generate a standardised MI-JSON file to improve front end technologies, in particular data visualisation.

## Conclusions

JAMI is proving its value as a framework to remove the need for redundant software development and testing with every release of a new or updated molecular interaction data standard. We intend to continue its development, extend its functionality and make it applicable to a wider set of use cases. The further development of JAMI is an open source project coordinated through the ‘MICommunity’ GitHub organisation (https://github.com/MICommunity) with documentation available at https://github.com/MICommunity/psi-jami/tree/master/docs and also code examples at https://github.com/MICommunity/psi-jami/tree/master/jami-examples. Please join us if you are interested in its future development.

## Availability and requirements

Project name: PSI-MI JAMI library Project home page: e.g. http://psidev.info/groups/molecular-interactions, https://github.com/MICommunity/psi-jami

Operating system(s): Platform independent.

Programming language: Java.

Other requirements:

License: Apache2.0.

Any restrictions to use by non-academics: None.

## Abbreviations

HUPO: Human Proteomics Organization
IMEx Consortium: International Molecular Exchange Consortium
JAMI: Java Molecular Interaction framework
MI: Molecular Interactions
PSI: Proteomics Standards Initiative
